# Schizophrenia Adoption Studies

**DOI:** 10.1371/journal.pmed.0030366

**Published:** 2006-08-29

**Authors:** Jonathan Leo

**Affiliations:** Lincoln Memorial University, Harrogate, Tennessee, United States of America

Patrick Sullivan cites twin and adoption studies as justification for searching for schizophrenia genes [1]. In his words, “Both adoption and twin studies indicate that the familiality of schizophrenia is due mainly to genetic effects.” To support this he provides a table (Table 1 in [1]) briefly summarizing these studies. Under “Adoption,” he mentions, “Adoptees with schizophrenia: increased risk in biological vs. adoptive parents (OR = 5.0; 95% CI 2.4–10.4).” However, rather than making a comparison between biological and adoptive parents, the original investigators made comparisons between index biological relatives and control biological relatives.

In the schizophrenia literature three studies have used this design; the lead author of all three was Seymour Kety (1968, 1975, and 1994). All three studied people who grew up as adopted children and were later diagnosed with a “schizophrenia spectrum disorder.” The goal was to examine the rate of schizophrenia spectrum disorders among those adoptees' biological family members (with whom they did not grow up) and compare that rate to the rate among the biological relatives of control adoptees, who were not diagnosed with a schizophrenia spectrum disorder [2].

According to Kety himself, a comparison between index adoptees' biological and adoptive relatives is “improper” and “fallacious” [3,4]. Indeed, in Kety's first adoption study (1968) there was no significant elevation of chronic schizophrenia, or of schizophrenia spectrum disorders, among his index biological versus index adoptive relatives. (This is based on the data; the investigators did not make this comparison.)

In addition, Kety and his colleagues did not limit themselves to looking at only parents. If they had done so, their conclusions would have been very different. For instance, in the 1975 study there were five index biological relatives diagnosed with chronic schizophrenia, but four of these five were half-siblings. The other diagnosis was given to a biological parent. In the 1968 Kety study, not a single index biological parent was diagnosed with chronic schizophrenia.

In his conclusion, Sullivan says that the treatment of the mentally ill mirrors the humanity of a society. True, but it then becomes difficult to rationalize the treatment of schizophrenia in this country in light of the World Health Organization studies showing that doctors in developing countries use less medication yet have a higher success rate than doctors in America. This is well documented in Robert Whitaker's book *Mad in America: Bad Medicine, Bad Science, and Enduring Mistreatment of the Mentally Ill* [5].
